# Plasma total antioxidant status in horses after 8-hours of road transportation

**DOI:** 10.1186/1751-0147-55-58

**Published:** 2013-08-14

**Authors:** Artur Niedźwiedź, Krzysztof Kubiak, Józef Nicpoń

**Affiliations:** 1 Department of Internal Diseases with Clinic for Horses, Dogs and Cats, The Faculty of Veterinary Medicine, Wroclaw University of Environmental and Life Sciences, pl. Grunwaldzki 47, 50-366, Wrocław, Poland

**Keywords:** Horse, Road transport, Oxidative stress, Antioxidants, Plasma total antioxidant status

## Abstract

**Background:**

The aim of this study was to investigate the effects of 8-hour road transport on plasma total antioxidant status (PTAS) and general clinical appearance in horses.

**Findings:**

The study was conducted on a group of 60 horses of different breeds aged from 4 to 10 years. Venous blood was collected and a clinical examination was performed immediately before loading horses onto trailers for an 8 hour transport (I), immediately after unloading them from the trailer (II), and after a subsequent 24 hour stall rest (III). The ferric-reducing ability of plasma (FRAP) was used to determine PTAS. The transportation significantly increased respiratory and heart rates. The average PTAS increased during the three subsequent samplings: I: 170 ± 77 (μmol/l) II: 204 ± 70 (μmol/l) III: 221 ± 74 (μmol/l).

**Conclusion:**

Long-distance transport increased the PTAS horses, as well as respiratory and heart rates.

## Findings

Many horses are transported every day for reasons such as shows, competitions, slaughter, exhibitions, breeding, hospitalization, and leisure [1,2]. Most horses are transported by truck and are often well adapted to road travels. However, transport can be a very stressful for horses being unfamiliar with travel [3,4]. Some of the strongest stressors associated with transport include the loading and unloading from trucks, penning in an unknown environment, confinement with and without motion, vibrations, changes in temperature and humidity, inadequate ventilation, and, sometimes deprivation of food and water [5]. Behavioral indicators of stress are vocalization, attempts to escape, kicking and struggling [6] and some studies have documented the adverse effects of transportation stress on horses [7,8]. However, to the authors’ knowledge, there are few studies concerning the influence of transport on the oxidative/antioxidative balance of horses [9-11]. To investigate the total antioxidant status (TAS), researchers have assessed a number of markers of oxidative stress and antioxidant defense. The TAS reflect the antioxidant level of antioxidant enzymes and nonenzymatic compounds including albumin, bilirubin and uric acid (endogenous antioxidant components) [12,13] as well as exogenous antioxidants such as carotenoids, vitamin E, ascorbic acid and flavonoids. The TAS has been developed to determine the synergistic role of all enzymatic as well as nonenzymatic antioxidants, rather than using the simple sum of individual antioxidants [14,15].

The purpose of this study was to investigate the effect of 8-hour road transport on the plasma total antioxidant status (PTAS) in horses and the horse’s general clinical appearance.

This study was conducted with the approval of the 2^nd^ Local Ethics Committee on Animal Experimentation in Wrocław (resolution No 39/2007). The animals included 60 cold-blooded horses intended for slaughter based on decisions made by individual owners. The ages of the horses ranged from 4 to 10 years, with 28 geldings and 32 mares. The mean ± SD weight was 680 ± 50 kg. The horses were typically fed twice a day (at 06.00 a.m. and 06.00 p.m.) with 2 kg of hay, approximately 1 kg of crushed oat and were given water *ad libitum*. On the morning of each road transport, 20 animals were loaded onto a livestock trailer at 9 am and transported 560 km. This procedure was repeated twice, and 20 horses were transported in each consecutive week. The transport route encompassed all types of road but the majority of the route was spent on national asphaltic roads. The transporter was driven at an average speed of 70 km/h (range 50–80). The study was conducted in November, and the mean daily outside air temperature and relative humidity were 4.5 ± 2.7°C and 93 ± 4.9%, respectively. Data on temperature and relative humidity inside the trailer during transport were not available.

The floor of the transporter was bedded with straw for each journey. During transit, the animals had access to hay, which was placed in hay nets and was accessible to each horse, but water was not available. During the 24 hour period in the abattoir after unloading from the transporter, horses had *ad libitum* water access and 2 kg of hay were given once after they were installed in the abattoir stables.

The horses underwent a clinical examination before and directly after transport, which included standard clinical procedures. Blood was collected from the external jugular vein using a disposable 20 mL syringe and 20 gauge needle into 2 mL tubes containing K2-EDTA (Sarstedt, Nümbrecht, Germany). The clinical examination and blood sampling were carried out three times: I – immediately before transport to the abattoir (ca. 2 h after a meal), II - immediately after unloading the animals from the trailer, after a journey lasting approximately eight hours (access to hay only), III - after a 24 h rest at the stable of the abattoir (access to hay and water only). Blood samples were centrifuged at 600 g for 20 min at 4°C. Plasma samples were immediately divided into Eppendorf tubes and stored at -80°C until analysis. The ferric reducing ability of plasma (FRAP) method was chosen to assess the PTAS [16]. The FRAP assay determines the ability of the plasma to reduce ferric iron to ferrous iron in a low pH environment. A colored ferrous-tripyridyltriazine complex is formed during this process and has a maximum absorbance at 593 nm. A Technikon RA-XT™ (Technicon Instruments Corporation, USA) analyzer was used. The results are expressed as μmol Trolox per L plasma.

The conformity of variable distributions with the normal distribution was assessed using the Kolmogorov-Smirnov test. All parameters were found to be compatible with a normal distribution. Parametric comparisons were carried out using the ANOVA analysis of variance in combination with the Tukey test. A paired t-test was used to compare values between the three groups (I, II, III). Significance was set at *P* < 0.05.

Differences in mean PTAS were found for the different sampling times. The average PTAS relative to time increased during the three subsequent samplings (*P* < 0.05) (Table 1). Statistically significant differences were found between the first and second samplings, as well as between sampling I and III (*P* < 0.05).

**Table 1:** Plasma total antioxidant status (PTAS) for 60 horses before transport (I), after 8 hours of travel (II), and after 24 hours of subsequent rest (III)

**Marker (unit)**	**I **_**(n=60)**_	**II**_**(n=60)**_	**III**_**(n=60)**_	***P*-value**
**PTAS (μmol/l)**	170 ± 77cpe	204 ± 70	221 ± 74	*P* = 0.004

Values are expressed as mean ± SD. The PTAS level at time I differ significantly from times II and III.

Basic clinical parameters including the capillary refill time (CRT), rectal temperature, heart and respiratory rates were recorded for all horses (Table 2). The heart rate and the respiratory rate were significantly increased (*P* < 0.05) between the first and second examination. Alterations in the general condition of horses may be evaluated by physiological, biochemical and behavioral criteria. In our study, the heart rate and respiratory rate were significantly increased (*P* = 0.0169 and 0.0155 respectively), while the body temperature and CRT were not changed during transport or during the post-transport period. These observations are consistent with results of other authors, who studied the impact of road transport on basic clinical signs [17,18]. The respiration rate was increased in horses in response to a 12 hour transport period and the heart rate was increased in horses after a 24 h transport [7]. An increased respiration rate in horses after a 12 hour transport period suggests that animals were not trained or used to being transported and they probably suffered from physiological stress or a combination of both [4,19]. The return to baseline of the heart and respiratory rates after 24 hours of rest may indicate that animals adapted quickly to the new environment. Hydration and establishment of hierarchy in the flock have a major influence on the stabilization of the altered parameters [20,21].

**Table 2:** Clinical parameters before (I), after 8 h of transportation (II) and after 24 h resting (III)

**Clinical examination parameter**	**I**	**II**	**III**	***P*-value**
**Heart rate** (beats/min.)	32 ± 3.1	38.1 ± 3.3	35.6 ± 3.1	*P* = 0.0169
**Repsiratory rate** (cycle/min.)	13.1 ± 1.8	20.2 ± 2.8	13.9 ± 2	*P* = 0.0155
**Rectal temperature**	37.6 ± 0.4	37.8 ± 0.5	37.4 ± 0.4	NS
(°C)				
**Capillary refill time**	1–2	1–2	1–2	NS
(sec.)				

Values are expressed as mean ± SD based on 60 horses. Heart rate at time I differ significantly from times II and III. Respiratory rate at time I differ significantly from time II.

In the present study, PTAS measured by the FRAP assay was significantly higher in the second and third samplings compared to levels before transport. This increase was probably related to an increase in the plasma concentrations of small molecule antioxidants that quickly redistribute to the blood. These substances include bilirubin, uric acid and albumin. Considering the decrease in the activity of antioxidant enzymes and increased levels of specific small molecule antioxidants, it can be concluded that the increase of the PTAS in transported horses was the result of the intensification of free radical processes in the body, as a result of acting stressors, as it was shown in cattle [11].

A statistically significant increase in the concentration of total protein and albumin was found in this study (data not shown). Increased concentration of these compounds is usually associated with transient dehydration [22]. However, there was no evidence of dehydration and/or electrolyte disturbances. Taking into account that the horses had constant access to water after unloading, we hypothesize that the significantly increased levels of total protein and albumin may be related to the antioxidant properties of blood proteins, particularly albumin, as a result of an increased production of reactive oxygen species as previously shown [22]. Our findings clearly differ from other studies that have demonstrated a decreased PTAS and increased lipid peroxidation in horses subjected to long-distance transport [9,15]. Moreover, stress caused by transportation significantly decreased the PTAS and significantly increase serum malondialdehyde concentrations in beef cattle [7]. Virtually all studies dealing with oxidative stress in horses during road transport focus on basic laboratory findings, activity of enzymatic antioxidants or lipid peroxidation products, while studies on oxidative stress in horses mainly deals with exercise physiology in Thoroughbreds and other sport horses [22,23].

A significant increase of the PTAS in transported horses may suggest a significant influence of non-enzymatic antioxidants on homeostatic mechanisms in equines. The estimation of the direct relationship between physiological stress and road transport on free radical processes in horses during transport needs further examinations.

Long-distance transport increased the PTAS in horses, as well as respiratory and heart rates and it can be concluded that PTAS the can provide a better understanding of the stress reaction and metabolic processes in transported horses.

## Competing interests

The authors declare that they have no competing interests.

## Authors’ contributions

AN and KK designed the study and did the laboratory work. JN coordinated the writing and editing of the manuscript. All authors read and approved the final manuscript.
